# Effects of ABCB1 gene polymorphisms on autonomic nervous system activity during atypical antipsychotic treatment in schizophrenia

**DOI:** 10.1186/s12888-018-1817-5

**Published:** 2018-07-17

**Authors:** Saki Hattori, Akira Suda, Ikuko Kishida, Masatoshi Miyauchi, Yohko Shiraishi, Mami Fujibayashi, Natsuki Tsujita, Chie Ishii, Norio Ishii, Toshio Moritani, Masataka Taguri, Yoshio Hirayasu

**Affiliations:** 1 Department of Psychiatry, Yokohama City University School of Medicine, 3-9 Fukuura, Kanazawa-ku, Yokohama, Kanagawa 236-0004 Japan; 2 Fujisawa Hospital, 383 Kotuka, Fujisawa, Kanagawa 251-8530 Japan; 3 Division of Physical and Health Education, Setsunan University, 17-8 Ikedanakamachi, Neyagawa, Osaka 572-8508 Japan; 4 Graduate School of Human and Environmental Studies, Kyoto University, Yoshidanihonmatsucho, Sakyo-ku, Kyoto, 606-8316 Japan; 5 Faculty of General Education, Kyoto Sangyo University, Kamo-motoyama, Kita-ku, Kyoto, 606-8555 Japan; 6 Department of Biostatistics, Yokohama City University School of Medicine, 3-9 Fukuura, Kanazawa-ku, Yokohama, Kanagawa 236-0004 Japan; 7 Hirayasu Hospital, 346 Kyozuka, Urasoe, Okinawa 901-2553 Japan

**Keywords:** Schizophrenia, Atypical antipsychotic drug, Heart rate variability, P-glycoprotein, Autonomic nervous system, ABCB1 gene

## Abstract

**Background:**

There are interindividual differences in the adverse effects of atypical antipsychotics, which include autonomic nervous system (ANS) dysfunction. Accordingly, to clarify the interindividual differences in the adverse effects of specific atypical antipsychotics in schizophrenia, we investigated the association between ANS dysfunction and ATP-binding cassette transport sub-family B member 1 (ABCB1) gene polymorphisms in patients with schizophrenia.

**Methods:**

In total, 233 Japanese patients with schizophrenia participated in this study. All of the participants received an atypical antipsychotic as monotherapy: 89 participants received risperidone, 69 olanzapine, 48 aripiprazole, and 27 quetiapine. ANS activity was assessed by means of a power spectral analysis of heart rate variability. Four single nucleotide polymorphisms (SNPs) in ABCB1 (rs1045642, rs1128503, rs2032582, and rs2235048) were genotyped using the TaqMan method.

**Results:**

For aripiprazole, sympathetic and total autonomic nervous activities were significantly lower in the rs1045642 T allele carrier–rs2235048 C allele carrier group than in the rs1045642 non-T allele carrier–rs2235048 non-C allele carrier group. In addition, in the aripiprazole group, the T-C-T-A haplotype (rs1045642-rs2235048-rs1128503-rs2032582) was associated with decreased ANS activity. However, there were no significant associations between ANS activity and ABCB1 gene polymorphisms in the risperidone, olanzapine, and quetiapine groups. Multiple regression analysis revealed that sympathetic and total nervous activities were significantly associated with the ABCB1 rs1045642–rs2235048 genotype and the T-C-T-A haplotype (rs1045642-rs2235048-rs1128503-rs2032582).

**Conclusion:**

We suggest that ABCB1 genetic polymorphisms affect aripiprazole-related ANS dysfunction but do not affect risperidone-, olanzapine-, or quetiapine-related ANS dysfunction.

## Background

Autonomic nervous system (ANS) activity is lower in patients with schizophrenia than in the general population [1, 2]. In patients with schizophrenia, antipsychotic medication is known to exacerbate ANS dysfunction [3–9], and reduced ANS activity is associated with cardiovascular morbidity and sudden death [10]. We previously reported that antipsychotic drugs significantly decrease ANS activity in a dose-dependent manner [8] and that risperidone, olanzapine, quetiapine, and aripiprazole, all atypical antipsychotics, exert several effects on ANS activity [11]. We found individual differences in ANS activity in patients with schizophrenia, even among those taking the same atypical antipsychotic (risperidone, olanzapine, aripiprazole, or quetiapine). Thus, in this study, we examined the pharmacogenetic interindividual differences in the effects of particular atypical antipsychotics, with an emphasis on the relationship with the activity of P-glycoprotein (P-gp), to clarify the individual differences in ANS activity during atypical antipsychotic―risperidone, olanzapine, quetiapine, and aripiprazole―treatment in schizophrenia.

P-gp, which is encoded by ATP-binding cassette transport sub-family B member 1 (ABCB1) and is also known as multidrug resistance protein 1 (MDR1), is expressed in various tissues, including the brain, kidney, liver, and intestine. P-gp functions as an important efflux pump in the blood-brain barrier for many kinds of drugs, including antipsychotics, and influences the brain availability of antipsychotics by transporting them against a concentration gradient through the blood-brain barrier and reducing their storage in the brain. Four atypical antipsychotics―risperidone, olanzapine, quetiapine, and aripiprazole―are substrates of P-gp [12–14]. Thus, P-gp activity within the blood-brain barrier in humans may affect antipsychotic tolerability [13, 14]. In a previous study, we examined the difference in the effects of these four atypical antipsychotics on ANS activity in patients with schizophrenia [11]. Other studies reported that ABCB1 gene polymorphisms affect the plasma concentrations of antipsychotics [15, 16]. Furthermore, ABCB1 gene polymorphisms affect the treatment response or adverse effects to antipsychotics such as weight gain or QTc prolongation [17–22]. We also previously reported associations of ABCB1 gene polymorphisms with ANS dysfunction following treatment with aripiprazole in patients with schizophrenia [23]. However, few studies have investigated the effects of ABCB1 gene polymorphisms on ANS dysfunction due to several other atypical antipsychotics in schizophrenia.

Accordingly, in this study, we investigated the association between ABCB1 genetic polymorphisms and ANS dysfunction in patients with schizophrenia treated with four atypical antipsychotic drugs—risperidone, olanzapine, quetiapine, and aripiprazole—to clarify whether ABCB1 genetic polymorphisms alter their adverse effects. We aimed to identify the biological factors affecting the antipsychotic response, specifically ANS dysfunction.

## Methods

### Participants

This cross-sectional study involved 233 consecutive Japanese patients with schizophrenia (94 men and 139 women; age [mean ± standard deviation], 51.88 ± 15.49 years) who attended Fujisawa Hospital and Yokohama City University Hospital in Japan. We recruited patients with schizophrenia who had received risperidone, olanzapine, quetiapine, or aripiprazole monotherapy for more than 3 months. No antipsychotic agents had been altered in the preceding 3 months. Participants who could not take the antipsychotics as prescribed and those who had not received hospital treatment for at least 1 year were excluded. Diagnosis was made by Japanese board certified psychiatrists with appropriate experience in clinical psychiatry, based on the Diagnostic and Statistical Manual of Mental Disorders, 4th edition criteria [24]. Participants with cardiovascular, respiratory, neurological, or endocrine illness; those with a current or past history of substance abuse that affected diagnosis; and those who were receiving medication for physical illness were excluded.

We assessed the severity of symptoms using a Japanese translation of the Positive and Negative Syndrome Scale (PANSS) [25]. The participants’ positive, negative, and general signs were evaluated by experienced clinical psychiatrists with the PANSS on the day of electrocardiography (ECG).

Clinical details were obtained from medical records for all participants. We investigated all psychotropic medications given, including benzodiazepines, and antipsychotic and antiparkinsonian agents. Doses were calculated with the appropriate conversion to the standard equivalent of chlorpromazine, biperiden, and diazepam (1 mg chlorpromazine = 0.5 mg clozapine) [26].

This two-center study was conducted in Fujisawa Hospital and Yokohama City University Hospital in Japan, with approval by the ethics committee of Fujisawa Hospital and in accordance with the Declaration of Helsinki. All participants provided written informed consent to participate in the study.

### R-R interval power spectral analysis

To evaluate ANS activity, a computer-assisted measurement of the 5-min resting heart rate variability (HRV) was performed, as in our previous work [27, 28]. HRV power spectral analysis can be applied to noninvasively assess autonomic imbalance by detecting the three major spectral components of HRV and has been used in basic medical science and clinical studies under various psychophysiological conditions. Its reliability, validity, and practicability have been proven [29–31]. Experiments were carried out in the mornings between 9:00 am and 12:00 pm. On the morning of the day of measurement, participants were required to abstain from ingesting caffeine or smoking tobacco. ECG was performed with the patients seated for 5 min after a rest of at least 10-min beforehand. HRV power spectral analysis uses the fast Fourier transform algorithm for decomposition of a series of sequences of R–R intervals acquired from the 5-min ECG into the sum of several sinusoidal functions of varying amplitudes and frequencies [27, 28, 32–35]. We quantified spectral power in the frequency domain by calculating the area under the curve in the following frequency bands, as we have previous reported [27–31]: low-frequency (LF; 0.03–0.15 Hz) HRV, indicative of both sympathetic and parasympathetic activity; high-frequency (HF; 0.15–0.40 Hz) HRV, indicative of primarily parasympathetic activity; and total power (TP; 0.03–0.40 Hz), indicative of total ANS activity [32–34].

### DNA extraction, ABCB1 SNP selection, and genotyping

Peripheral blood was collected from all patients, and DNA was extracted for genotype determination. Four SNPs—rs1045642, rs1128503, rs2032582, and rs2235048—were selected from the NCBI dbSNP database. We focused on these particular SNPs because rs1045642 and rs2032582 have been implicated in marked changes in P-gp expression and plasma drug levels [36]. Furthermore, these four SNPs are associated with hyperprolactinemia and other antipsychotic drug-related adverse effects [17, 21, 37]. Genotype analysis was performed by genomic DNA extraction from peripheral blood leukocytes using these ABCB1 gene SNPs (rs1045642, rs1128503, rs2032582, and rs2235048). Information on the SNPs is shown in Table 1. Analysis of all SNPs was performed by using TaqMan SNP genotyping assay with the ABI Prism 7900HT Sequence Detection System [38]. Haplotype frequency was determined by using the Haploview program (version 4.1).

**Table 1:** The ABCB1 gene and associated polymorphisms

SNP	Region	Allele
rs1045642	Chr.7: 87509329	C/T
rs1128503	Chr.7: 87550285	C/T
rs2032582	Chr.7: 87531302	G/A•T
rs2235048	Chr.7: 87509195	T/C

### Statistical analysis

All statistical analyses were carried out with SPSS for Windows (version 24; SPSS, Chicago, IL). The Student’s *t*-test was used to compare the LF, HF, and TP components of HRV according to genotype in the two patient groups. The Student’s *t*-test was also used to compare demographic and medication data between the rs1045642 C–rs2235048 T allele carrier group and the rs1045642 non-C–rs2235048 non-T allele carrier group. Using multiple regression analysis, we also assessed the effects of rs1045642–rs2235048 genotype and clinical factors on ANS activity. Dependent variables were the HRV components LF, HF, and TP; independent variables that could influence ANS activity were age, body mass index, PANSS, and ABCB1 genetic polymorphisms [11, 39]. Linkage disequilibrium was analyzed using Haploview software (version 4.1) [40]. HRV spectral component absolute values were log-transformed before statistical analysis because of the presence of skewed data. A value of *p* < .05 was considered statistically significant.

## Results

The demographic and medication data and HRV components of all participants are shown in Table 2. There were 89 participants in the risperidone group (34 men and 55 women; age [mean ± standard deviation], 54.85 ± 14.11 years; CPZ [the daily doses of antipsychotic drugs were converted to an approximate chlorpromazine equivalent], 397.47 ± 224.24 mg/day), 69 participants in the olanzapine group (25 men and 44 women; 51.51 ± 15.87 years; 472.46 ± 227.44 mg/day), 48 participants in the aripiprazole group (24 men and 24 women; 45.13 ± 13.78 years; 465.10 ± 191.14 mg/day), and 27 participants in the quetiapine group (11 men and 16 women; 55.07 ± 18.44 years; 490.63 ± 306.11 mg/day). Table 3 shows the genotypic and allelic frequencies of the four polymorphisms and the HRV in each atypical antipsychotic group. Significant deviation from the expected Hardy–Weinberg proportions were not seen in any of the SNPs. Haplotype analysis revealed perfect linkage disequilibrium between ABCB1 rs1045642 and rs2235048 (r^2^ = 1). The aripiprazole group had significantly lower HRV LF and TP components in the rs1045642 T allele carrier–rs2235048 C allele carrier group versus the rs1045642 non-T allele carrier–rs2235048 non-C allele carrier group (LF, *p* = .008; TP, *p* = .017; Table 3). These patients also showed a tendency toward a lower HRV HF component in the rs1045642 T allele carrier–rs2235048 C allele carrier group versus the rs1045642 non-T allele carrier–rs2235048 non-C allele carrier group (*p* = .097). There were no significant differences between all components of HRV and the ABCB1 gene polymorphisms―rs1045642, rs1128503, rs2032582, and rs2235048―in the risperidone, olanzapine, and quetiapine groups. In addition, regarding the association between haplotypes and ANS activity, T-C-T-A haplotype carriers (rs1045642-rs2235048-rs1128503-rs2032582) had significantly lower HRV components versus the non-T-C-T-A haplotype carriers in the aripiprazole group (LF, *p =* .005; HF, *p* = .036; TP, *p* = .008). Table 4 shows the demographic and medication data of both the rs1045642 T allele carrier–rs2235048 C allele carrier group and the rs1045642 non-T allele carrier–rs2235048 non-C allele carrier group in the aripiprazole group. There were no significant differences in age, sex, PANSS total score, and medication data among the groups.

**Table 2:** Demographic and medication data and log-transformed power values of the LF, HF, and TP frequency bands of all participants

	All subjects (*n* = 233)
Age (years)	51.88 ± 15.49
Sex (male/female)	94/139
Inpatient/outpatient	56/177
Duration of illness (years)	20.21 ± 14.44
Smoking (smoker/non-smoker)	17/216
BMI (kg/m^2^)	24.08 ± 4.47
CPZeq^a^ (mg/day)	444.40 ± 231.41
BPDeq^b^ (mg/day)	0.83 ± 1.55
DZPeq^c^ (mg/day)	5.84 ± 8.71
PANSS total score	71.59 ± 15.55
lnLF (ms^2^)	4.37 ± 1.22
lnHF (ms^2^)	3.92 ± 1.45
lnTotal Power (ms^2^)	4.99 ± 1.21

Data are presented as the mean ± standard deviation

*BMI* body mass index, *PANSS* Positive and Negative Syndrome Scale, *ln* natural log-transformed, *HF* high frequency, *LF* low frequency, *TP* total power

^a^The daily doses of antipsychotic drugs were converted to an approximate chlorpromazine equivalent

^b^The daily doses of anticholinergic antiparkinsonian drugs were converted to an approximate biperiden equivalent

^c^The daily doses of benzodiazepine were converted to an approximate diazepam equivalent

**Table 3:** Associations between the ABCB1 genotype and ANS activity

	rs1045642			rs1128503			rs2032582			rs2235048		
	C/C	C/T, T/T	*P*	T/T	C/T, C/C	*P*	G/G	G/A•T A•T/A•T	*P*	T/T	C/C, C/T	*P*
Risperidone (*n* = 89)												
*N*	31	58		37	52		14	75		31	58	
lnLF (ms^2^)	4.38 ± 1.16	4.51 ± 1.25	.629	4.40 ± 1.15	4.51 ± 1.20	.693	4.59 ± 1.02	4.44 ± 1.26	.675	4.38 ± 1.16	4.51 ± 1.25	.629
lnHF (ms^2^)	3.94 ± 1.38	3.99 ± 1.68	.881	4.02 ± 1.62	3.94 ± 1.55	.831	4.35 ± 1.16	3.91 ± 1.63	.339	3.94 ± 1.38	3.99 ± 1.68	.881
lnTP (ms^2^)	5.00 ± 1.13	5.10 ± 1.31	.721	5.05 ± 1.29	5.08 ± 1.23	.898	5.26 ± 0.96	5.03 ± 1.26	.529	5.00 ± 1.13	5.10 ± 1.31	.721
Olanzapine (*n* = 69)												
*N*	20	49		35	34		15	54		20	49	
lnLF (ms2)	4.15 ± 1.22	4.40 ± 1.09	.421	4.41 ± 1.02	4.22 ± 1.23	.489	4.19 ± 1.06	4.36 ± 1.15	.605	4.15 ± 1.22	4.40 ± 1.09	.421
lnHF (ms2)	3.92 ± 1.23	3.98 ± 1.12	.851	3.98 ± 1.05	3.95 ± 1.25	.926	3.82 ± 1.48	4.00 ± 1.04	.581	3.92 ± 1.23	3.98 ± 1.12	.851
lnTP (ms2)	4.88 ± 1.05	5.05 ± 0.93	.511	5.06 ± 0.90	4.95 ± 1.05	.630	4.89 ± 1.07	5.04 ± 0.95	.601	4.88 ± 1.05	5.05 ± 0.93	.511
Aripiprazole (*n* = 48)												
*N*	17	31		17	31		9	39		17	31	
lnLF (ms^2^)	5.25 ± 0.93	4.30 ± 1.23	.008^a^	4.53 ± 1.38	4.69 ± 1.13	.665	5.14 ± 1.21	4.52 ± 1.20	.168	5.25 ± 0.93	4.30 ± 1.23	.008^a^
lnHF (ms^2^)	4.61 ± 1.40	3.87 ± 1.47	.097	4.40 ± 1.13	3.99 ± 1.51	.372	4.61 ± 1.51	4.02 ± 1.47	.287	4.61 ± 1.40	3.87 ± 1.47	.097
lnTP (ms^2^)	5.79 ± 1.05	4.90 ± 1.25	.017^a^	5.26 ± 1.35	5.19 ± 1.21	.851	5.69 ± 1.29	5.10 ± 1.23	.211	5.79 ± 1.05	4.90 ± 1.25	.017^a^
Quetiapine (*n* = 27)												
*N*	5	22		14	13		2	25		5	22	
lnLF (ms^2^)	3.85 ± 1.47	3.67 ± 1.30	.790	3.37 ± 1.31	4.07 ± 1.25	.170	4.18 ± 0.97	3.67 ± 1.34	.602	3.85 ± 1.47	3.67 ± 1.30	.790
lnHF (ms^2^)	4.60 ± 1.64	3.09 ± 1.57	.237	3.14 ± 1.71	3.41 ± 1.52	.672	5.20 ± 0.27	3.11 ± 1.56	.075	4.60 ± 1.64	3.09 ± 1.57	.237
lnTP (ms^2^)	4.70 ± 1.52	4.21 ± 1.38	.486	4.02 ± 1.49	4.61 ± 1.26	.350	5.53 ± 0.46	4.20 ± 1.39	.199	4.70 ± 1.52	4.21 ± 1.38	.486

Data are presented as the mean ± standard deviation

*ln* natural log-transformed, *HF* high frequency, *LF* low frequency, *TP* total power

^a^Significant difference (*P* < .05; Student’s *t*-test)

**Table 4:** Demographic and medication data between the rs1045642 C–rs2235048 T allele carrier group and the rs1045642 Non-C–rs2235048 Non-T allele carrier group in participants treated with aripiprazole monotherapy

	rs1045642 T/T, C/T rs2235048 C/C, C/T	rs1045642 C/C rs2235048 T/T	*P*
Age (years)	46.74 ± 14.03	42.18 ± 13.21	.277
BMI (kg/m^2^)	25.41 ± 5.32	24.43 ± 4.43	.625
PANSS total score	71.32 ± 15.33	72.82 ± 17.83	.761
Disease duration (years)	16.10 ± 13.34	16.94 ± 10.66	.823
CPZeq^a^ (mg/day)	445.97 ± 210.37	500.00 ± 149.48	.354
BPDeq^b^ (mg/day)	1.13 ± 2.78	0.47 ± 0.87	.348
DZPeq^c^ (mg/day)	3.93 ± 5.47	6.89 ± 7.50	.124

Data are presented as the mean ± standard deviation

*BMI* body mass index, *PANSS* Positive and Negative Syndrome Scale

^a^The daily doses of antipsychotic drugs were converted to an approximate chlorpromazine equivalent

^b^The daily doses of anticholinergic antiparkinsonian drugs were converted to an approximate biperiden equivalent

^c^The daily doses of benzodiazepine were converted to an approximate diazepam equivalent

Multiple regression analysis revealed a significant association between the LF and TP components of HRV and the ABCB1 rs1045642–rs2235048 genotype (LF, *p* = .009; TP, *p* = .024; Table 5) after adjustment for age, which affects HRV [11]. There were no significant associations of all components of HRV with BMI or PANSS in the multiple regression analysis. Regarding the aripiprazole group, a significant association was found between the HRV LF and TP components and the T-C-T-A haplotype (rs1045642-rs2235048-rs1128503-rs2032582) (LF, *p* = .003; TP, *p* = .010); the HRV HF component showed a tendency for an association with the T-C-T-A haplotype (rs1045642-rs2235048-rs1128503-rs2032582) (*p* = .067) in the multiple regression analysis.

**Table 5:** Multiple regression analysis results using ANS activity, age, body mass index, PANSS, and type of ABCB1 rs1045642–rs22235048 genotype as independent variables in participants treated with aripiprazole monotherapy

Independent variable	ANS activity					
	lnLF		lnHF		lnTP	
	Β	*P*	β	*P*	β	*P*
Age (years)	−.043	<.001^a^	−.037	.018^a^	−.0410	.001^a^
BMI (kg/m^2^)	.042	.145	.026	.534	.0348	.277
PANSS (total score)	−.001	.925	−.014	.253	−.006	.494
Type of rs1045642–rs2235048 genotype (reference category: T/C allele carrier)	.798	.009^a^	.618	.148	.745	.024^a^

*ANS* autonomic nervous system, *BMI* body mass index; *ln* natural log-transformed, *HF* high frequency, *LF* low frequency, *PANSS* Positive and Negative Syndrome Scale, *TP* total power

^a^Significant difference (*P* < .05; multiple regression analysis)

## Discussion

To our knowledge, this study is the first in which the associations between ABCB1 gene polymorphisms―rs1045642, rs1128503, rs2032582, and rs2235048―and adverse effects, specifically ANS dysfunction, in patients with schizophrenia were compared among atypical antipsychotics. We newly found differences in ANS dysfunction according to ABCB1 genetic polymorphism in schizophrenic patients treated with aripiprazole. On the other hand, there were no significant differences in ANS dysfunction according to ABCB1 genetic polymorphism in schizophrenic patients treated with risperidone, olanzapine, or quetiapine. In terms of ABCB1 rs104542 and rs2235048, the rs1045642 T allele carrier–rs2235048 C allele carrier group had significantly lower LF and TP components of HRV compared with the rs1045642 non-T allele carrier–rs2235048 non-C allele carrier group in the aripiprazole group. This implies an association of the T allele of rs1045642 and the C allele of rs2235048 with reduced sympathetic activity in aripiprazole-treated schizophrenia patients.

ABCB1 rs1045642 at position 3435 on exon 26 does not alter the amino acid sequence [41] but is associated with changes in the expression of P-gp [36, 42]. The rs1045642 T variant is linked to decreased intestinal expression of MDR1 and lower plasma levels of digoxin, which is a substrate of P-gp [36, 42]. Additionally, ABCB1 rs1045642 and rs2032582 affect plasma aripiprazole levels [43]. Systemic pharmacological inhibition of ABCB1 also results in elevated brain levels of nelfinavir, the ABCB1 substrate, even with only marginal changes in plasma levels [44]. In addition, ABCB1 polymorphisms have been found to be associated with an increased concentration of the drug in the cerebrospinal fluid [45]. Regarding a clinical study of adverse effects related to rs1045642, Suzuki et al. reported that the QT interval was significantly longer in patients with schizophrenia treated with risperidone with the rs1045642 T allele than in those with the rs1045642 non-T allele [20]. Accordingly, we believe that the rs1045642 T variant might also alter the brain levels of aripiprazole and centrally affect sympathetic activity in schizophrenic patients treated with aripiprazole, although this is a matter of speculation.

ABCB1 rs2235048 is localized to the 27th intron of the 3′ end of the gene and, although it is a synonymous SNP, it may influence the structure of P-gp [46]. ABCB1 rs2235048 has been associated with the efficacy of risperidone and paliperidone in schizophrenia [37]. However, there are no other studies regarding the association between rs2235048 and the antipsychotic response in schizophrenia.

rs2235048 and rs1045642 are less than 200 bp apart and showed complete linkage disequilibrium in the patients in this study. In fact, a previous study reported that the T > C polymorphism of rs2235048 is associated with low activity of the T allele at rs1045642 [47]. A low-activity T allele at rs1045642 reduces ABCB1 splicing efficiency, which would lower the rate of production of correctly spliced RNA, and hence P-gp, in low-activity T homozygotes [36]. We suggest that rs2235048 affects ANS activity by altering the activity of P-gp, particularly in rs1045642 individuals.

Kirschbaum et al. reported that the significant increase in the brain-to-serum concentrations of both aripiprazole and its metabolite dehydroaripiprazole, in particular, after their acute and subchronic administration was related to the expression of P-gp, as indicated by higher levels in ABCB1ab double knockout mice [13]. In addition, Nagasaka et al. reported that aripiprazole and dehydroaripiprazole have higher inhibition potencies for P-gp activity than risperidone, paliperidone, olanzapine, and ziprasidone [48]. Rafaniello et al. reported that ABCB1 genetic polymorphisms affected plasma aripiprazole levels [43]. On the other hand, no associations have been reported between ABCB1 polymorphisms, including rs1128503, rs2032582, and rs1045642, and plasma levels of quetiapine, risperidone, and 9-hydroxyrisperidone [49, 50]. In addition, olanzapine has lower affinity for P-gp than risperidone or quetiapine [12]. Thus, the tolerability and response of aripiprazole could be more strongly affected by ABCB1 gene polymorphisms than those of the other atypical antipsychotics, and ABCB1 gene polymorphisms were only associated with aripiprazole-related ANS activity and not with risperidone-, olanzapine-, or quetiapine-related ANS activity.

In this study, there were significant associations between the ABCB1 haplotype and aripiprazole-related ANS activity. In schizophrenic patients treated with aripiprazole, the T-C-T-A haplotype (rs1045642-rs2235048-rs1128503-rs2032582) was linked to reduced ANS activity. Previous studies reported that inhibition potency to P-gp was significantly different between ABCB1 T-T-A haplotype (rs1045642-rs1128503-rs2032582) carriers and ABCB1 haplotype non-carriers during treatment with methadone, which is a substrate of P-gp [51]. In addition, tremor due to antipsychotics significantly differed between C-G-C haplotype carriers (rs1045642-rs20325820-rs1128503) and non-carriers [37]. The blood prolactin increase due to antipsychotics was also significantly associated with ABCB1 T-A-T haplotype carriers (rs1045642-rs20325820-rs1128503) [37]. Therefore, we believe that the ABCB1 haplotype might affect P-gp activity and drug response to a greater extent than a single ABCB1 gene polymorphism. Thus, the ABCB1 haplotype could affect aripiprazole brain availability and the aripiprazole response, including ANS activity.

There were no significant differences in the demographic data, symptom severity by PANSS, and medication data between the rs1045642 T allele carrier–rs2235048 C allele carrier group and the rs1045642 non-T allele carrier–rs2235048 non-C allele carrier group for the patients receiving aripiprazole. In addition, multiple regression analysis revealed that sympathetic and total nervous system activity was significantly associated with the ABCB1 rs1045642–rs2235048 genotype and the ACBC1 T-C-T-A haplotype. Although multiple regression analysis also revealed an association between age and both sympathetic and parasympathetic nervous system activity, there was no significant difference in age between the ABCB1 rs1045642 T allele carrier–rs2235048 C allele carrier and rs1045642 non-C allele carrier–rs2235048 non-T allele carrier groups, as mentioned above.

Our study has some limitations. Casual relationships could not be determined due to the cross-sectional design. The sample size was small, particularly that of the quetiapine group. We could not investigate the serum and brain concentrations of the antipsychotics. Although all participants received antipsychotic monotherapy, we did not eliminate the effects of other classes of medications that might influence ANS activity. In addition, we did not investigate QTc interval prolongation, and future studies should clarify the association between ANS dysfunction and QTc interval prolongation related to ABCB1 gene polymorphisms. Because it remains unclear whether ANS activity induces cardiovascular events, we need to follow and monitor the course of ABCB1 rs1045642 T allele carriers–rs2235048 C allele carriers who are under treatment with aripiprazole.

## Conclusions

In conclusion, our findings indicate that ABCB1 genetic polymorphisms affect aripiprazole-related ANS activity but do not affect risperidone-, olanzapine-, or quetiapine-related ANS activity. We suggest that the ABCB1 rs1045642 T allele and rs2235048 C allele decrease sympathetic and total nervous activity system in patients with schizophrenia treated with aripiprazole and that ABCB1 rs1045642 and rs2235048 and the ABCB1 haplotype (rs1045642-rs2235048-rs1128503-rs2032582) could affect the tolerability of aripiprazole and aripiprazole-induced ANS dysfunction. Our results suggest that clinicians should bear in mind a higher risk of ANS dysfunction in patients with schizophrenia who are ABCB1 rs1045642 T allele carriers and rs2235048 C allele carriers and under aripiprazole treatment. Studies with a larger sample size are necessary to elucidate the effects of antipsychotics on ANS activity in schizophrenic patients.

## Abbreviations

ABCB1: ATP-binding cassette transport sub-family B member 1
ANS: Autonomic nervous system
BMI: Body mass index
BPD: Biperiden
CPZ: Chlorpromazine
DZP: Diazepam
ECG: Electrocardiography
HF: High frequency
HRV: Heart rate variability
LF: Low frequency
ln: Natural log-transformed
MDR1: Multidrug resistance protein 1
PANSS: Positive and Negative Syndrome Scale
P-gp: P-glycoprotein
SNP: Single nucleotide polymorphism
TP: Total power

## References

[1] Clamor A, Lincoln TM, Thayer JF, Koenig J (2016). Resting vagal activity in schizophrenia: meta-analysis of heart rate variability as a potential endophenotype. Br J Psychiatry.

[2] Quintana DS, Westlye LT, Kaufmann T, Rustan OG, Brandt CL, Haatveit B, Steen NE, Andreassen OA (2016). Reduced heart rate variability in schizophrenia and bipolar disorder compared to healthy controls. Acta Psychiatr Scand.

[3] Agelink MW, Majewski T, Wurthmann C, Lukas K, Ullrich H, Linka T, Klieser E (2001). Effects of newer atypical antipsychotics on autonomic neurocardiac function: a comparison between amisulpride, olanzapine, sertindole, and clozapine. J Clin Psychopharmacol.

[4] Birkhofer A, Geissendoerfer J, Alger P, Mueller A, Rentrop M, Strubel T, Leucht S, Forstl H, Bar KJ, Schmidt G (2013). The deceleration capacity – a new measure of heart rate variability evaluated in patients with schizophrenia and antipsychotic treatment. Eur Psychiatry.

[5] Cohen H, Loewenthal U, Matar M, Kotler M (2001). Association of autonomic dysfunction and clozapine. Heart rate variability and risk for sudden death in patients with schizophrenia on long-term psychotropic medication. Br J Psychiatry.

[6] Huang WL, Chang LR, Kuo TB, Lin YH, Chen YZ, Yang CC (2013). Impact of antipsychotics and anticholinergics on autonomic modulation in patients with schizophrenia. J Clin Psychopharmacol.

[7] Ieda M, Miyaoka T, Wake R, Liaury K, Tsuchie K, Fukushima M, Araki T, Ezoe S, Inagaki T, Horiguchi J (2014). Evaluation of autonomic nervous system by salivary alpha-amylase level and heart rate variability in patients with schizophrenia. Eur Arch Psychiatry Clin Neurosci.

[8] Iwamoto Y, Kawanishi C, Kishida I, Furuno T, Fujibayashi M, Ishii C, Moritani T, Taguri M, Hirayasu Y (2012). Dose-dependent effect of antipsychotic drugs on autonomic nervous system activity in schizophrenia. BMC Psychiatry.

[9] Kim JH, Yi SH, Yoo CS, Yang SA, Yoon SC, Lee KY, Ahn YM, Kang UG, Kim YS (2004). Heart rate dynamics and their relationship to psychotic symptom severity in clozapine-treated schizophrenic subjects. Prog Neuro-Psychopharmacol Biol Psychiatry.

[10] Thayer JF, Yamamoto SS, Brosschot JF (2010). The relationship of autonomic imbalance, heart rate variability and cardiovascular disease risk factors. Int J Cardiol.

[11] Hattori S, Kishida I, Suda A, Miyauchi M, Shiraishi Y, Fujibayashi M, Tsujita N, Ishii C, Ishii N, Moritani T, Taguri M, Hirayasu Y (2018). Effects of four atypical antipsychotics on autonomic nervous system activity in schizophrenia. Schizophr Res.

[12] Boulton DW, DeVane CL, Liston HL, Markowitz JS (2002). In vitro P-glycoprotein affinity for atypical and conventional antipsychotics. Life Sci.

[13] Kirschbaum KM, Uhr M, Holthoewer D, Namendorf C, Pietrzik C, Hiemke C, Schmitt U (2010). Pharmacokinetics of acute and sub-chronic aripiprazole in P-glycoprotein deficient mice. Neuropharmacology.

[14] Wang JS, Zhu HJ, Donovan JL, Yuan HJ, Markowitz JS, Geesey ME, Devane CL (2009). Aripiprazole brain concentration is altered in P-glycoprotein deficient mice. Schizophr Res.

[15] Suzuki Y, Tsuneyama N, Fukui N, Sugai T, Watanabe J, Ono S, Saito M, Someya T (2013). Impact of the ABCB1 gene polymorphism on plasma 9-Hydroxyrisperidone and active moiety levels in Japanese patients with schizophrenia. J Clin Psychopharmacol.

[16] Suzuki T, Mihara K, Nakamura A, Kagawa S, Nagai G, Nemoto K, Kondo T (2014). Effects of genetic polymorphisms of CYP2D6, CYP3A5, and ABCB1 on the steady-state plasma concentrations of aripiprazole and its active metabolite, dehydroaripiprazole, in Japanese patients with schizophrenia. Ther Drug Monit.

[17] Bozina N, Kuzman MR, Medved V, Jovanovic N, Sertic J, Hotujac L (2008). Associations between MDR1 gene polymorphisms and schizophrenia and therapeutic response to olanzapine in female schizophrenic patients. J Psychiatr Res.

[18] Furukori Y, Tsuchimine S, Saito M, Nakagami T, Sato Y, Kaneko S (2007). Association between major multidrug resistance 1 (MDR1) gene polymorphisms and plasma concentration of prolactin during risperidone treatment in schizophrenic patients. Prog Neuro-Psychopharmacol Biol Psychiatry.

[19] Kuzman MR, Medved V, Bozina N, Grubisin J, Jovanovic N, Sertic J (2011). Association study of MDR1 and 5-HT2C genetic polymorphisms and antipsychotic-induced metabolic disturbances in female patients with schizophrenia. The Pharmacogenomics Journal.

[20] Suzuki Y, Tsuneyama N, Fukui N, Sugai T, Watanabe J, Ono S, Saito M, Inoue Y, Someya T (2014). Effect of risperidone metabolism and P-glycoprotein gene polymorphism on QT interval in patients with schizophrenia. The Pharmacogenomics Journal.

[21] Xing Q, Gao R, Li H, Feng G, Xu M, Duan S, Meng J, Zhang A, Qin S, He L (2006). Polymorphisms of the ABCB1 gene are associated with the therapeutic response to risperidone in Chinese schizophrenia patients. Pharmacogenomics.

[22] Xu Q, Wu X, Li M, Huang H, Minica C, Yi Z, Wang G, Shen L, Xing Q, Shi Y, He L, Qin S (2016). Association studies of genomic variants with treatment response to risperidone, clozapine, quetiapine and chlorpromazine in the Chinese Han population. The Pharmacogenomics Journal.

[23] Hattori S, Suda A, Kishida I, Miyauchi M, Shiraishi Y, Fujibayashi M, Tsujita N, Ishii C, Ishii N, Moritani T, Taguri M, Hirayasu Y. Associations of ABCB1 gene polymorphisms with aripiprazole-induced autonomic nervous system dysfunction in schizophrenia. Schizophrenia Resarch. 2017; 10.1016/j.schres.2017.11.020. 10.1016/j.schres.2017.11.020 29191720

[24] American Psychiatric Association (1994). Diagnostic and Statistical Manual of Mental Disorders, fourth ed.

[25] Kay SR, Fiszbein A, Ople LA (1987). The positive and negative syndrome scale (PANSS) for schizophrenia. Schizophr Bull.

[26] Inada T, Inagaki A (2015). Psychotropic dose equivalence in Japan. Psychiatry Clin Neurosi.

[27] Fujibayashi M, Matsumoto T, Kishida I, Kimura T, Ishii C, Ishii N, Moritani T (2009). Autonomic nervous system activity and psychiatric severity in schizophrenia. Psychiatry Clin Neurosci.

[28] Kimura T, Matsumoto T, Akiyoshi M, Owa Y, Miyasaka N, Aso T, Moritani T (2006). Body fat and blood lipids in postmenopausal women are related to resting autonomic nervous system activity. Eur J Appl Physiol.

[29] Matsumoto T, Miyawaki T, Ue H, Kanda T, Zenji C, Moritani T (1999). Autonomic responsiveness to acute cold exposure in obese and non-obese young women. Int J Obes Relat Metab Disord.

[30] Matsumoto T, Ushiroyama T, Morimura M, Moritani T, Hayashi T, Suzuki T, Tatsumi N (2006). Autonomic nervous system activity in the late luteal phase of eumenorrheic women with premenstrual symptomatology. J Psychosom Obstet Gynaecol.

[31] Moritani T, Kimura T, Hamada T, Nagai N (2005). Electrophysiology and kinesiology for health and disease. J Electromyogr Kinesiol.

[32] Akselrod S, Gorden D, Ubel FA, Shannon DC, Berger AC, Cohen RJ (1981). Power spectrum analysis of heart of fluctuation: a quantitative probe of beat-to-beat cardiovascular control. Science.

[33] Pagani M, Lombardi F, Guzzeti S, Rimoldi O, Furlan R, Pizzinelli P, Sandrone G, Malfatto G, Dell’Orto S, Piccaluga E (1986). Power spectral analysis of heart rate and arterial pressure variabilities as a marker of sympatho-vagal interaction in man and conscious dog. Circ Res.

[34] Pomeranz B, Macaulay RJ, Caudill MA, Kutz I, Adam D, Gordon D, Kilborn KM, Barger AC, Shannon DC, Cohen RJ (1985). Assessment of autonomic function in humans by heart rate spectral analysis. Am J Phys.

[35] Rompelman O, Coenen AJ, Kitney RI (1977). Measurement of heart-rate variability: part 1-comparative study of heart-rate variability analysis methods. Med Biol Eng Comput.

[36] Hoffmeyer S, Burk O, von Richter O, Arnold HP, Brockmoller J, Johne A, Cascorbi I, Gerloff T, Roots I, Eichelbaum M, Brinkmann U (2000). Functional polymorphisms of the human multidrug-resistance gene: multiple sequence variations and correlation of one allele with P-glycoprotein expression and activity in vivo. Proc Natl Acad Sci U S A.

[37] Mi W, Liu F, Liu Y, Du B, Xiao W, Li L, Huang L, Lu T, He J, Shi L, Yue W, Zhang H (2016). Association of ABCB1 gene polymorphisms with efficacy and adverse reaction to risperidone or Paliperidone in Han Chinese schizophrenic patients. Neurosci Bull.

[38] Applied Biosystems, Foster City, Ca, USA.

[39] Wang J, Liu YS, Zhu WX, Zhang FQ, Zhou ZH (2014). Olanzapine-induced weight gain plays a key role in the potential cardiovascular risk: evidence from heart rate variability analysis. Sci Rep.

[40] Barrett JC, Fry B, Maller J, Daly MJ (2005). Haploview: analysis and visualization of LD and haplotype maps. Bioinformatics.

[41] Gottesman MM, Hrycyna CA, Shoenlein PV, Germann UA, Pastan I (1995). Genetic analysis of the multidrug transporter. Annu Rev Genet.

[42] Sakaeda T, Nakamura T, Horinouchi M, Kakumoto M, Ohmoto N, Sakai T, Morita Y, Tamura T, Aoyama N, Hirai M, Kasuga M, Okumura K (2001). MDR1 genotype-related pharmacokinetics of digoxin after single oral administration in healthy Japanese subjects. Pharm Res.

[43] Rafaniello C, Sessa M, Bernardi FF, Pozzi M, Cheli S, Cattaneo D, Baldelli S, Molteni M, Bernardini R, Rossi F, Clementi E, Bravaccio C, Radice S, Capuano A. The predictive value of ABCB1, ABCG2, CYP3A4/5 and CYP2D6 polymorphisms for risperidone and aripiprazole plasma concentrations and the occurrence of adverse drug reactions. Pharmacogenomics J. 2017; 10.1038/tpj.2017.38. 10.1038/tpj.2017.38 28719598

[44] Choo EF, Leake B, Wandel C, Imamura H, Wood AJ, Wilkinson GR, Kim RB (2000). Pharmacological inhibition of P-glycoprotein transport enhances the distribution of HIV-1 protease inhibitors into brain and testes. Drug Metab Dispos.

[45] Basic S, Hajnsek S, Bozina N, Filipcic I, Sporis D, Mislov D, Posavec A (2008). The influence of C3435T polymorphisms of ABCB1 gene on penetration of phenobarbital across the blood-brain barrier in patients with generalized epilepsy. Seizure.

[46] Kimchi-Sarfaty C, Oh JM, Kim IW, Sauna ZE, Calcagno AM, Ambudkar SV, Gottesman MMA (2007). "Silent" polymorphism in the MDR1 gene changes substrate specificity. Science.

[47] Soranzo N, Cavalleri GL, Weale ME, Wood NW, Depondt C, Marguerie R, Sisodiya SM, Goldstein DB (2004). Identifying candidate causal variants responsible for altered activity of the ABCB1 multidrug resistance gene. Genome Res.

[48] Nagasaka Y, Oda K, Iwatsubo T, Kawamura A, Usui T (2012). Effects of aripiprazole and its active metabolite dehydroaripiprazole on the activities of drug efflux transporters expressed both in the intestine and at the blood-brain barrier. Biopharm Drug Dispos.

[49] Kim KA, Joo HJ, Lee HM, Park JY (2014). Influence of ABCB1 and CYP3A5 genetic polymorphisms on the pharmacokinetics of quetiapine in healthy volunteers. Pharmacogenet Genoimcs.

[50] Vandenberghe F, Guidi M, Choong E, von GA, Conus P, Csajka C, Eap CB (2015). Genetics-based population pharmacokinetics and pharmacodynamics of risperidone in a psychiatric cohort. Clin Pharmacokinet.

[51] Hung CC, Chiou MH, Teng YN, Hsieh YW, Huang CL, Lane HY (2013). Functional impact of ABCB1 variants on interactions between P-glycoprotein and methadone. PLoS One.

